# On the relation between electrical and electro-optical properties of tunnelling injection quantum dot lasers

**DOI:** 10.1515/nanoph-2022-0693

**Published:** 2023-01-31

**Authors:** Vissarion Mikhelashvili, Lior Gal, Guy Seri, Sven Bauer, Igor Khanonkin, Ori Eyal, Amnon Willinger, Johann Reithmaier, Gadi Eisenstein

**Affiliations:** Electrical and Computer Engineering Department and Russell Berrie Nanotechnology Institute, Technion, Haifa, 32000, Israel; Technische Physik, Institute of Nanostructure Technologies and Analytics, Center of Interdisciplinary Nanostructure Science and Technology (CINSaT), University of Kassel, Heinrich-Plett-Str. 40, 34132 Kassel, Germany

**Keywords:** negative capacitance, quantum dot, tunnelling

## Abstract

We present a comprehensive study of the temperature dependent electronic and optoelectronic properties of a tunnelling injection quantum dot laser. The optical power-voltage (*P*_opt_–*V*) characteristics are shown to be correlated with the current-voltage (*I*–*V*) and capacitance-voltage (*C*–*V*) dependencies at low and elevated temperatures. Cryogenic temperature measurements reveal a clear signature of resonant tunnelling manifested in periodic responses of the *I*–*V* and *P*_opt_–*V* characteristics, which diminish above 60 K. The *C*–*V* characteristics reveal a hysteresis stemming from charging and de-charging of the quantum dots, as well as negative capacitance. The latter is accompanied by a clear peak that appears at the voltage corresponding to carrier clamping, since the clamping induces a transient-like effect on the carrier density. *C*–*V* measurements lead also to a determination of the dot density which is found to be similar to that obtained from atomic force microscopy. *C*–*V* measurements enable also to extract the average number of trapped electrons in each quantum dot which is 0.95. As the important parameters of the laser have signatures in the electrical and electro-optical characteristics, the combination serves as a powerful tool to study intricate details of the laser operation.

## 1 Introduction

The most fundamental limitation of dynamic properties in all semiconductor lasers is the optical gain nonlinearity [1]. The gain nonlinearity originates from various physical processes including spectral and spatial hole burning and most important, hot carrier injection to the laser oscillating state. Hot carrier injection can in principle be overcome using the concept of tunnelling injection (TI) [1–8] which makes use of a quantum well (QW) serving as a cold carrier reservoir that feeds the laser oscillating state via a tunnelling process. Bhattacharya et al. [3] reported a TI Quantum Dot (QD) laser with a bandwidth of approximately 22 GHz. TI also improves temperature characteristics of QD lasers as demonstrated by Klopf et al. [9].

In TI QD lasers, carrier injection from the reservoir is achieved by hybridization between the QW confined state and an excited state of the QDs [5–7] followed by fast relaxation to the QD ground state. The wide, inhomogeneously broadened spectrum of the QDs ensures that the QW confined state is always aligned with an excited state of some QD. A key requirement is that the transition energy of the QDs that hybridize is close to the gain peak where laser oscillation occurs. Otherwise, the TI processes become a loss mechanisms and hampers the laser performance [8].

This paper addresses the manner by which TI impacts the electrical and electro-optical properties of TI QD lasers. The voltage dependence of current (*I*–*V*), capacitance (*C*–*V*), and optical power (*P*_opt_–*V*) are found to be strongly correlated when tested over a wide range of temperatures, from 4 K to 290 K. The comprehensive study leads to several important findings. These include the discretization of the dot layers as well as direct evidence of resonant tunnelling both of which are observed, at low temperatures, in the current and optical power dependencies on voltage. *C*–*V* characteristics reveal hysteresis and negative capacitance which is accompanied by a peak corresponding to carrier clamping. The measured *C*–*V* enables also to determine the dot density which is similar to that obtained in atomic force microscopy. It also yields the average number of electrons trapped in each QD which is 0.95.

The combined electrical and electro-optical characteristics make for a powerful set of experimental tools for detailed understanding of carrier transport and radiative processes in TI QD and other types of semiconductor lasers.

## 2 Experimental results and discussion

**Figure 1:**
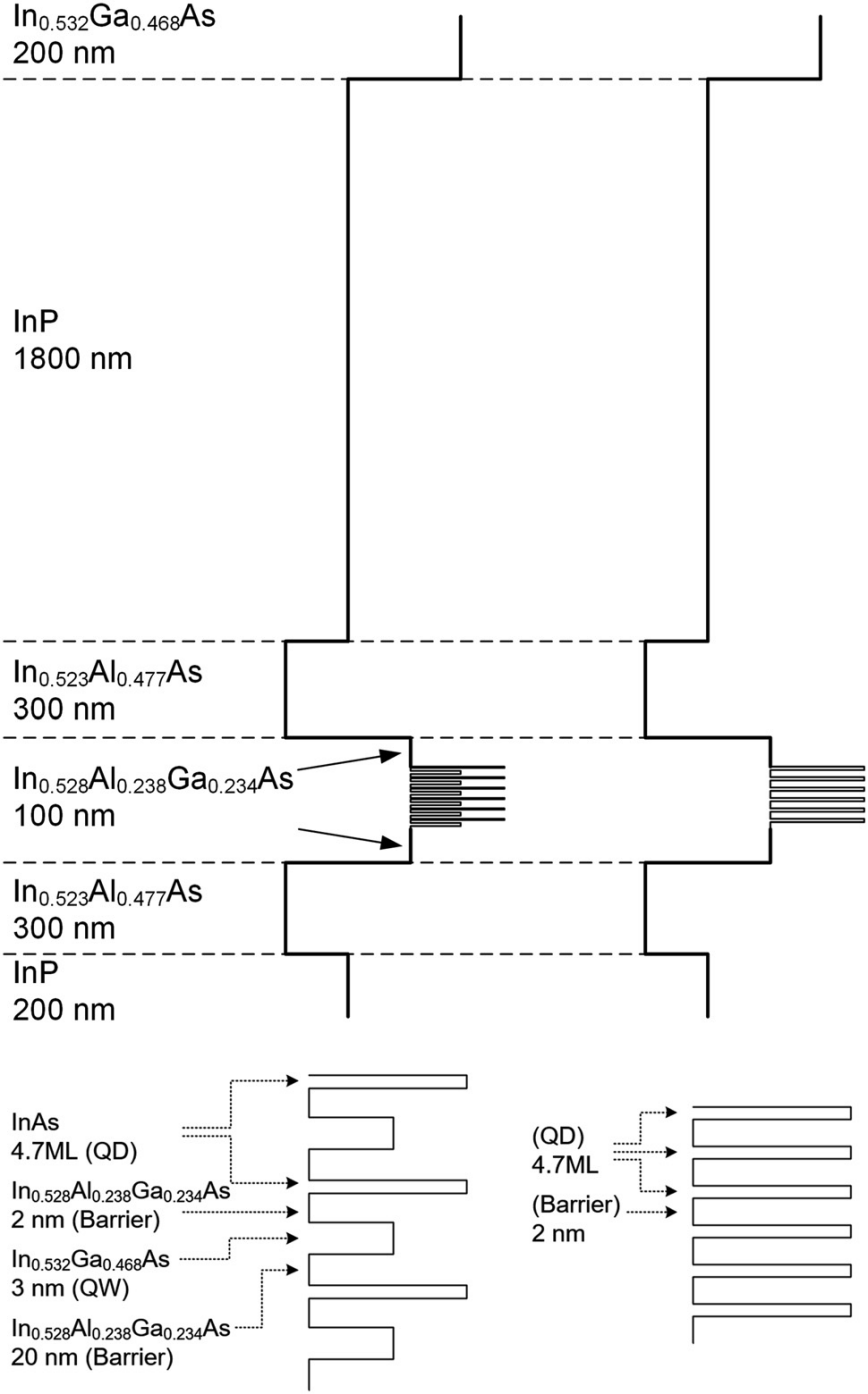
Energy band diagram of TI QD laser (left) and a conventional, reference QD laser (right). The lower parts are enlarged views of the respective active regions.

The TI QD laser comprised six layers of highly uniform InAs QDs with a density of 3 ⋅ 10^10^  cm^−2^. Each QD layer is accompanied by a 3 nm wide QW separated from the QDs by a 2 nm thick InAlGaAs layer. The homogeneity of the QD layers is characterized, by convention, via the photo luminescence line width at 10 K. For a single layer, the QDs exhibit a width of 17 meV while for a stack of 6 layers it is 35 meV [10]. These are narrower by a factor of 4–5 compared to any reported QDs in any material system. Figure 1 describes the energy band diagrams of the TI QD laser and a reference QD laser that has no TI region. An enlarged view of the active regions is shown in lower part of the figure. The laser cavity was formed by a 2 μm wide, 330 μm long ridge waveguide whose back facet reflectivity was larger than 90%, obtained by multi-layer dielectric coating comprising a three-layer stack of SiO_2_ and Si.

### 2.1 Current-voltage characteristics

**Figure 2:**
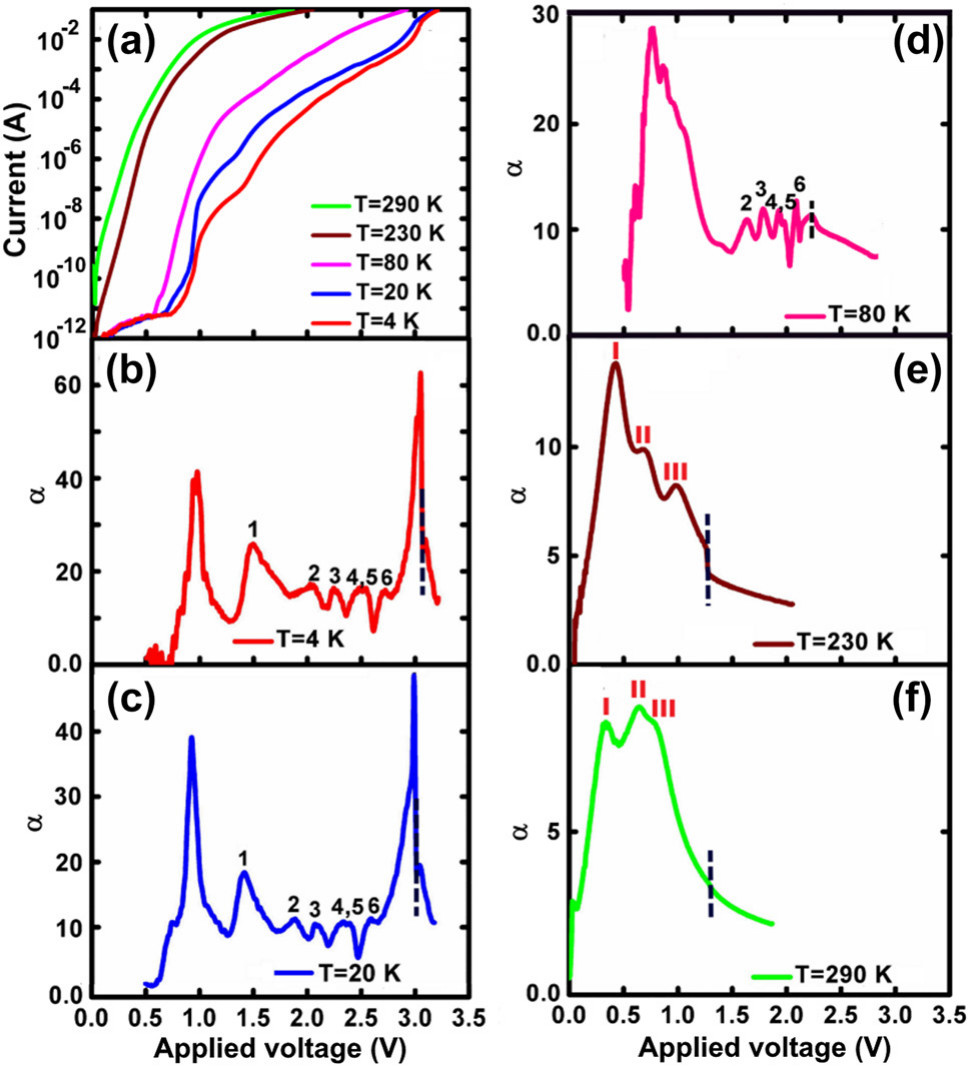
Temperature dependent (a) *I*–*V*, and (b)–(f) *α*–*V* characteristics. The dotted lines indicate the location of the corresponding voltages of the evolving discontinuity in Figure 2(b)–(f). Roman numerals mark the maxima and inflections points on the *α*–*V* curves.

Current-voltage characterizations, measured at different temperatures between 4 K and 290 K, are shown in Figure 2(a). The calculated power exponent parameter, $\alpha =d\left(\ln\;I\right)/d\left(\ln\;V\right)$ [11] dependence on bias (*α*–*V*) and temperature is shown in Figure 2(b)–(f).

At a very low forward bias, *α* exhibits a large peak that is associated with the diffusive component of the carrier flow from the cladding layers to the QW, which is highly temperature dependent. Following this large peak, at low temperatures, the *α*–*V* curve reveals an oscillatory nature which reflects discretization of the QD layers; there is one bump in the curve for each QD layer. At 3.05 V and at a temperature of 4 K, *α* exhibits a strong peak at the end of which appears a distinct discontinuity, which indicates carrier clamping at threshold [12]. Comparing the results with a reference QD laser having no TI region reveals a similar discretization at 4 K with the first large peak missing as there is no QW layer.

The bumps in the *α*–*V* curves are related to the step-like increment of the current with applied voltage. It is due to Coulomb blockade originating from the potential of fully charged dots in each layer, which opposes the applied bias, and also from a tunnelling process between separate QW–QD layer pairs. Each section of the *I*–*V* and *α*–*V* curves can be described by the theory of Asryan [13, 14] with the inclusion of various standard tunnelling mechanisms [15–18].

At elevated temperatures, the bumps in the *α*–*V* characteristics disappear since broadening of the homogeneous linewidth eliminates the discrete nature of the QD layers. A thermally activated branch of the *I*–*V* characteristic is dominated, at the lowest bias regime, by diffusion-recombination, in accordance with the classical Shockley-Reed-Hall model [19, 20]. At 230 K and 290 K, carrier transport is dominated by tunnelling at a somewhat increased voltage corresponding to the second and third peaks in Figure 2(e)–(f). This behavior results from the strong temperature dependence of the ideality factor and slope of the *I*–*V* characteristics, and the weak dependence of the saturation current [21].

**Figure 3:**
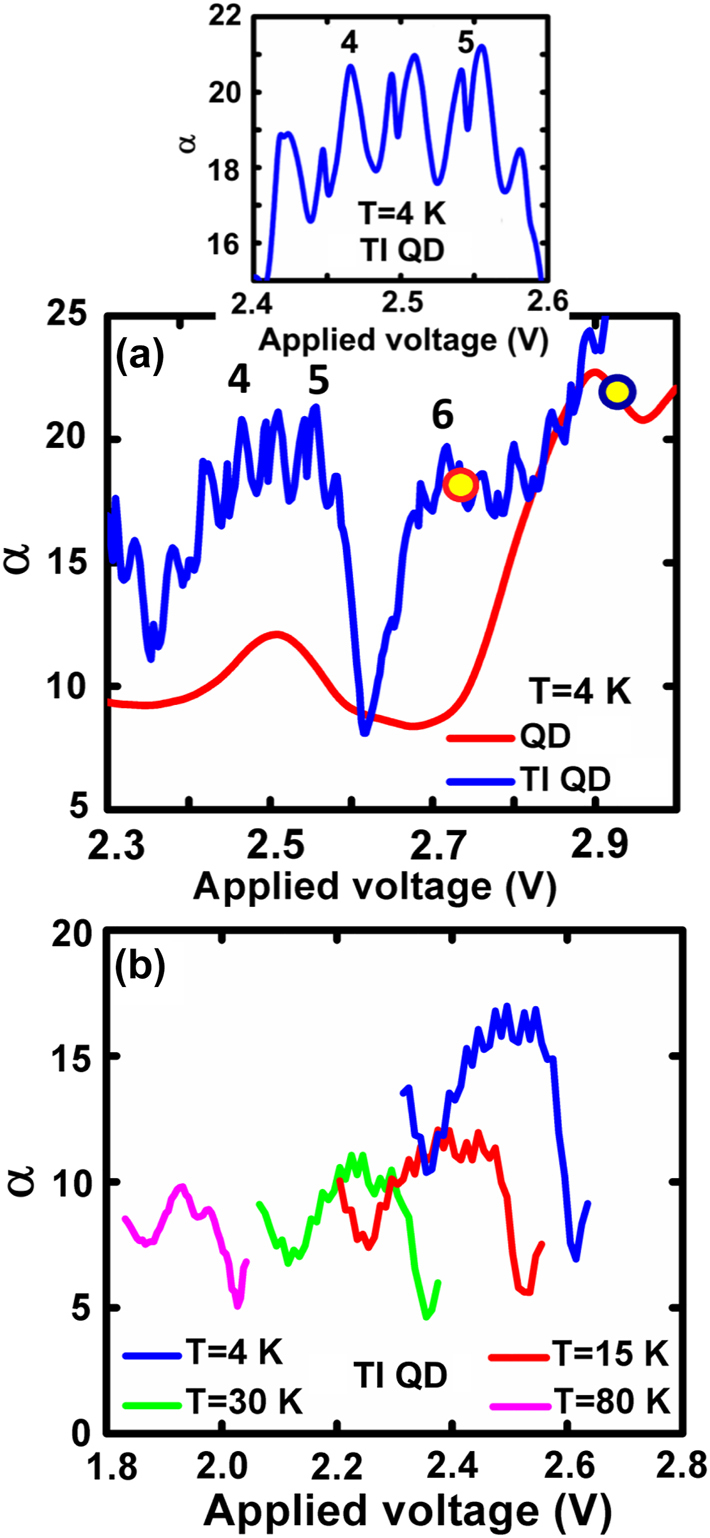
Temperature and bias dependent *α*. (a) High resolution *α*–*V* curves at 4 K measured with voltage steps of 1 mV for a TI QD laser and a conventional QD laser. The two yellow circles represent the two threshold voltages and the inset shows an enlarged view of the tunnelling resonances. (b) Evolution with temperature of the fourth and fifth bumps of the *α*–*V* curves measured with a voltage step of 10 mV.

A high-resolution *α*–*V* curve (with a 1 mV voltage step) measured at 4 K is shown in the blue trace of Figure 3(a) for the voltage range of 2.3 V–3 V. It reveals well-separated, periodic, narrow peaks which are superimposed on the bumps. These peaks are a direct imprint of the actual TI process, resembling classical resonant tunnelling. Since the QD ensemble is inhomogeneous, different voltages cause hybridization of excited states belonging to different QD groups (clusters) with the confined state of the QW injector [5–7], yielding successive resonant tunnelling events [22]. The *α*–*V* curve for a reference QD laser with no TI region is shown in the red trace of Figure 3(a) and naturally shows no resonances. The two yellow circles shown in Figure 3(a) represent the two respective threshold voltages.

**Figure 4:**
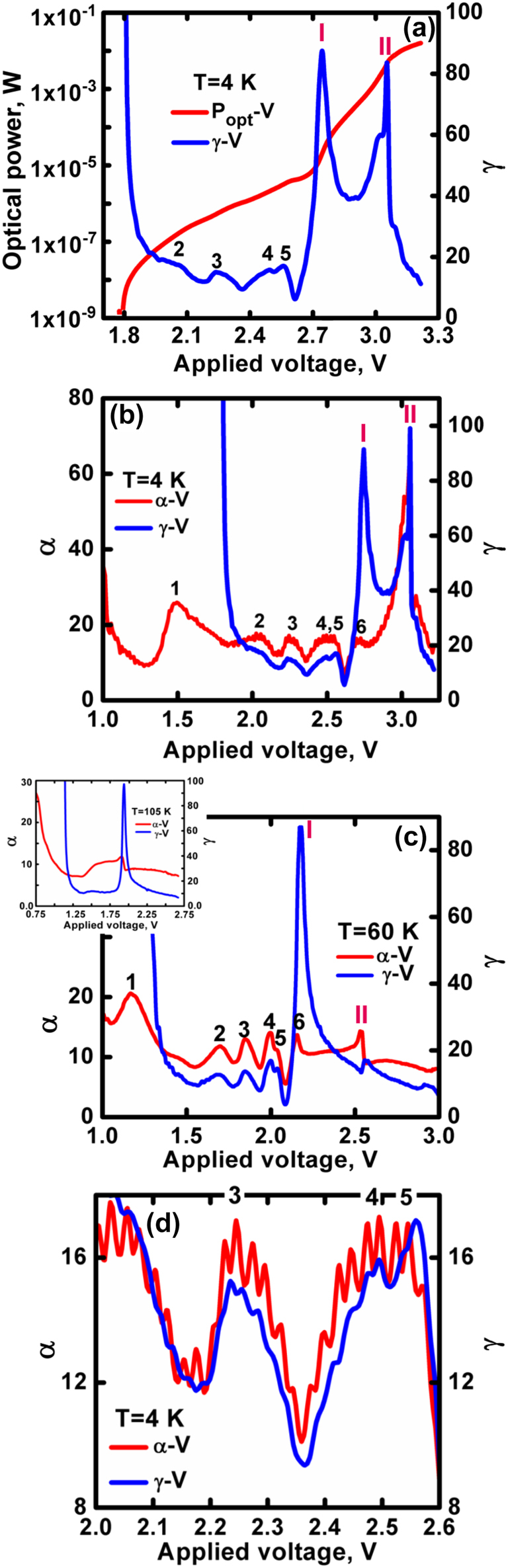
Temperature and bias dependent *α* and *γ*. (a) *P*_opt_–*V* and *γ*–*V* characteristics. Comparison of *γ*–*V* and *α*–*V* dependencies measured at (b) 4 K, and (c) 60 K. (d) High resolution comparison of *γ*–*V* and *α*–*V* showing an enlarged view of the third, fourth and fifth bumps. The inset in Figure 4(c) shows the *γ*–*V* and *α*–*V* characteristics at 105 K.

The periodic reiteration in Figure 3(a) does not exhibit single peaks, but rather double peaks making a W-like shape where the pairs of peaks are not symmetric relative to the central ones. This is highlighted in the inset of Figure 3(a). The distance between them is roughly 18 mV and 25 mV and their average full width at half maximum are 5.3 mV and 13.2 mV, respectively. The appearance of the W-like shaped peaks stems from the fact that they have a relatively wide energy span since the confined state of the injector QW can hybridize simultaneously with spectrally close excited states belonging to different groups of dots [6, 7] and therefore, resonant tunnelling occurs over a finite spectral range. For the same reason, the width of the peaks is wider than the common spectral width of a single dot at 4 K which is in the *μ*eV range. As the temperature increases, the *α*–*V* characteristics move to lower voltages and the resonance sharpness diminishes since the homogeneous linewidth of the QDs widens and the overlap between dot clusters increases. This is shown in Figure 3(b) for the fourth and fifth bumps where the resonances disappear at 80 K.

### 2.2 Power-voltage characteristics

The most basic electro-optical property of a diode laser is usually characterized by *P*_opt_–*I* measurements. *P*_opt_–*V* characteristics are not commonly used but they often show more intricate details and are easily correlated with the *I*–*V* characteristics [21]. The *P*_opt_–*V* characteristic measured at 4 K is described in Figure 4(a) together with the calculated *γ*–*V* characteristic, $\left(\gamma =d\left(\ln\;P_{\text{opt}}\right)/d\left(\ln\;V\right)\right)$ [21].

The QD layer discretization is also observed in the *γ*–*V* curve at 4 K which shows the same bumps as in the *α*–*V* curve except for the absence of the first peak since there is no emission of any kind at the very low bias voltage. The sixth bump is not seen and is replaced by a sharp peak (marked I) at 2.75 V which signifies the threshold voltage. The second large peak (marked II) at 3.05 V coincides with the peak in Figure 2(b). As the temperature rises, these two peaks evolve (see Figure 4(c)). Peak I shifts to the low voltage side, while peak II sharply decreases. Beyond 60 K they coincide and represent a single threshold voltage. This is shown in the inset to Figure 4(c) for 105 K. The main features of the low temperature *γ*–*V* characteristic, in particular the oscillating bumps follow the corresponding features of the *α*–*V* characteristic. This confirms the direct impact of the resonant tunnelling on the emission process. The oscillations superimposing the bumps in the *γ*–*V* characteristics are somewhat less pronounced compared to the *α*–*V* curve as seen in Figure 4(d). This is due to the fact that different dot layers contribute differently to the laser emission. Carrier transport effects and an internal field that bends the energy levels yield a non-uniform inversion [23].

**Figure 5:**
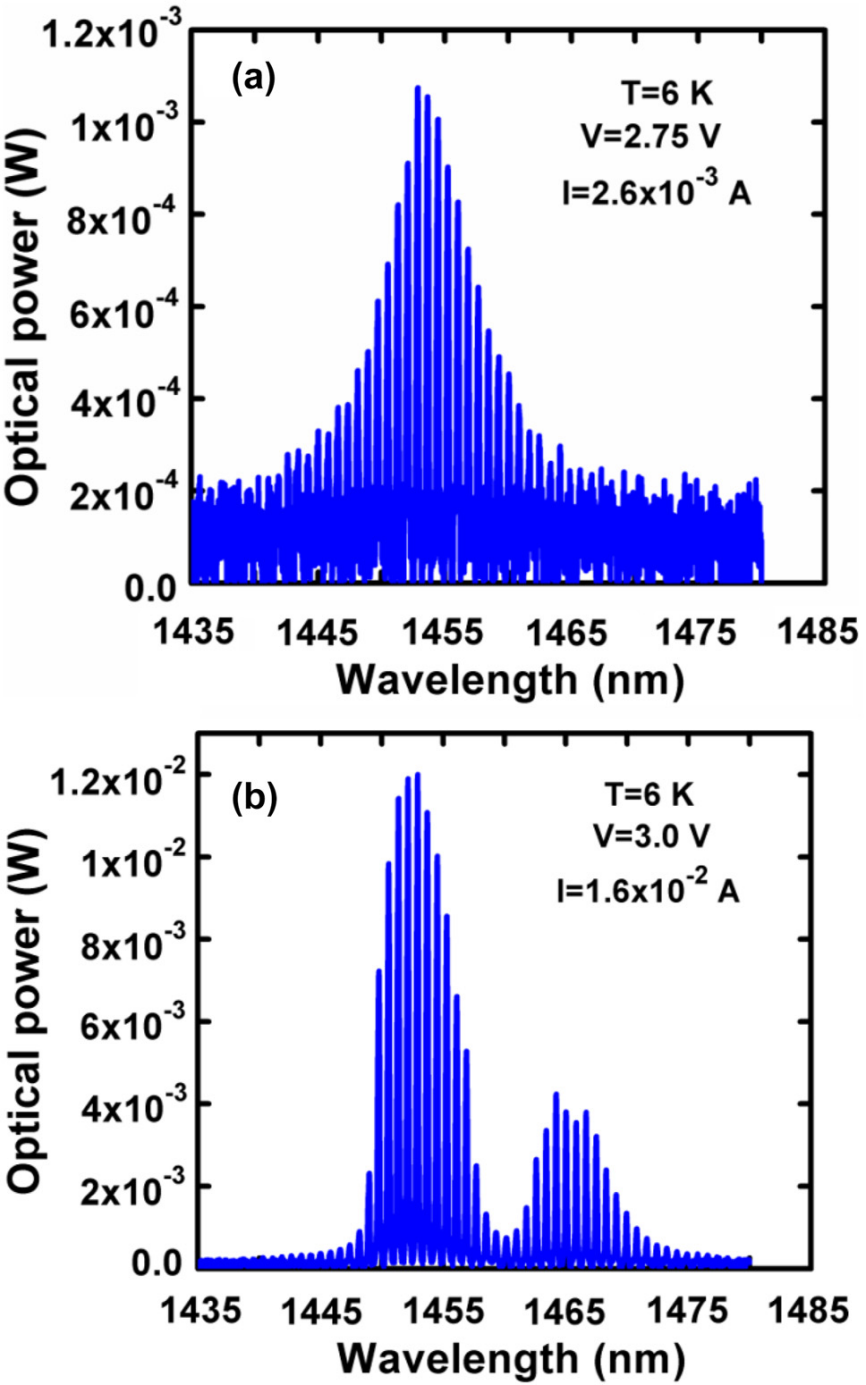
Applied voltage dependent optical spectra measured at 6 K.

The origin of the two peaks in the *γ*–*V* curve is the dot inhomogeneity which is highlighted at low temperatures due to narrowing of the dot’s homogeneous linewidth. This is seen in the bias dependent emitted spectra presented in Figure 5 for a temperature of 6 K.

At low bias levels (Figure 5(a)), the laser spectrum is that of a standard Fabry–Perot laser. As the bias increases, the spectrum becomes asymmetric and at about 3 V, the laser emits in two spectral regions as seen in Figure 5(b). This is a classical behavior of an inhomogeneously broadened laser [24]. The emergence of the second emitted spectral region coincides with the voltage of the second peak of the *γ*–*V* curve and the discontinuity in the *α*–*V* characteristic that is the voltage where carrier clamping takes place. As the temperature rises and the homogeneous linewidth increases, the discrete nature of the gain spectrum diminishes and the laser exhibits but one threshold. Correspondingly, the *γ*–*V* curve exhibits a single peak which coincides with the discontinuity in the *α*–*V* curve, which represents threshold.

### 2.3 Capacitance voltage characteristics

**Figure 6:**
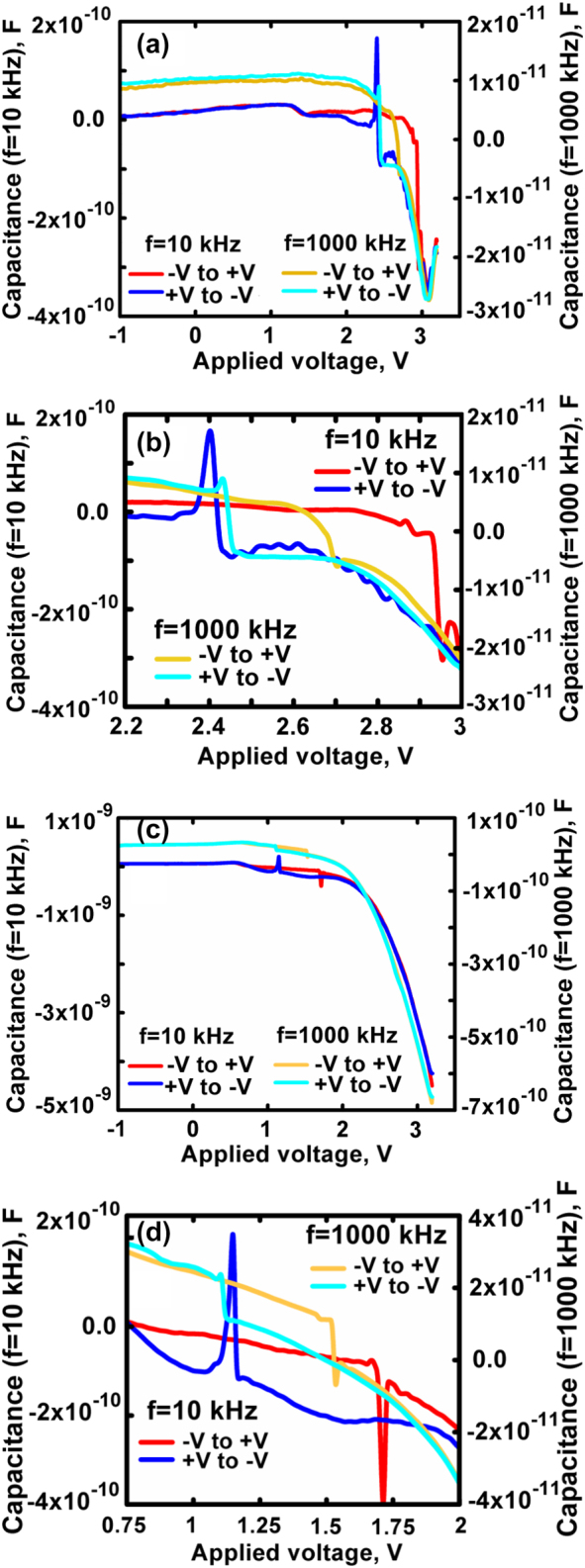
Temperature dependent *C*–*V* characteristics at two frequencies. (a) 10 K, (c) 150 K, (b) and (d), enlarged views at 10 K and 150 K, respectively, of the *C*–*V* curves near the hysteresis regions.

A full electrical analysis including its correlation to optoelectronic properties requires also a description of the *C*–*V* characteristics. Such measurements performed at 10 kHz and 1000 kHz are shown in Figure 6 for two temperatures. Under stimulated emission conditions, as the trapping and de-trapping processes in the QDs increase, the *C*–*V* characteristics exhibit an inductive like nature (known as negative capacitance (NC)) as often seen in light emitting diodes [25–28] and QD lasers [29].

The measured capacitance exhibits, for both frequencies, discontinuities at two different voltages what forms a clockwise hysteresis loop. It originates from charging of the QDs when the voltage varies from negative to positive and discharging for the opposite polarity. Figure 6(b) and (d) show enlarged views near the hysteresis region at 10 K and 150 K, respectively. The hysteresis loop formation resembles that of nonvolatile memory type metal–insulator–semiconductor structures or field effect transistors, with embedded metal or semiconductor nanocrystals [30, 31]. The hysteresis loop shifts towards negative bias values as the temperature changes from 10 K and 150 K.

The average QD density can be estimated by the width of the hysteresis loop which is determined, in turn, by the total sum of trapped charges in each QD [32]. Charges accumulated in the QDs induce, under bias, charges with the opposite sign in the semiconductor substrate, what shifts the capacitance to higher voltages. The InAs average dot density is estimated as [32, 33]:

$$n_{\mathrm{dot}}=\frac{\varepsilon_{0}\varepsilon_{\text{tun}}\Delta V_{C-V}}{q\nu \left(Nd_{\text{cnt}}+\frac{\varepsilon_{\text{tun}}}{2\varepsilon_{\text{InP}}}d_{QD}N_{QD}\right)}$$

The constants *ɛ*_0_ and *q* are, respectively, the vacuum permittivity and the elementary charge, while *ɛ*_tun_ = 13.4 and *ɛ*_InP_ = 12.4 are the respective dielectric coefficients of the tunnelling layer and the InP substrate layer. *d*_cnt_ = 2 nm is the barrier layer width between dot layers (playing the role of a control layer [32, 33]), *d*_QD_ = 1.38 nm (4.7 mono-layers) is the QD mean diameter. There are *N* = 5 control layers, and *N*_QD_ = 6 QD layers. We further evaluate the effective number of electrons captured by a single QD using $\nu =\frac{d_{\text{eff}}}{\Delta E_{g}}$ [30] where $\Delta E_{g}=\frac{q^{2}}{C_{\text{self}}}$ is the Coulomb energy gap and *C*_self_ = 2*πɛ*_0_ *ɛ*_tun_ *d*_QD_ is the self-capacitance. *d*_eff_ = *φ*_sub_ − *φ*_QD_ is the depth of the effective potential well of the QD region relative to substrate that depends on *φ*_sub_ = 4.65 eV [34] and *φ*_QD_ = 4.83 eV [35] which are the respective work functions of the InP substrate and InAs QD layer (the latter is similar to the work function value *φ*_InAs_ = 4.9 eV [36] for monocrystalline InAs). We obtain that *d*_eff_ = 0.18 eV, the Coulomb band gap is Δ*E*_*g*_ = 0.19 eV and thus *ν* = 0.95. Considering a hysteresis width of Δ*V*_*C*_–_*V*_ = 0.53 *V* (based on Figure 6), the average QD density per layer is found to be *n*_dot_ = 5.3 ⋅ 10^10^ cm^−2^ which is close to the estimated density of 3 ⋅ 10^10^ cm^−2^ obtained by atomic force microscope [10].

Under a high stimulated emission rate, carrier injection to the QDs is dominated by carriers that originate in the QW reservoir and feed the ground state via a hybrid state and Coulomb scattering. This leads to a fast recombination rate of the ground state electrons which are replenished by carriers that originate in the hybrid state and relax fast to the ground state via Coulomb scattering [5–7]. In addition to enhancing the radiative recombination, the carriers captured by the QDs, together with the injected holes, affect the capacitance and cause the hysteresis loop whose width is determined by the sum of charges trapped in each QD.

In the forward high-voltage regime, the measured capacitance is essentially the junction capacitance of a modified quasi-P-I-N structure with an effective thickness of its intrinsic layer being lower than at low bias levels. At low bias levels, the thickness comprises the six identical QW, barrier, and QD sublayers. Since the junction capacitance is $C=\frac{dQ}{dV_{j}}$ [37] (Q being the charge), and since the differential junction voltages *dV*_j_ in diodes with monotonically increasing *I*–*V* characteristics is always positive, the junction capacitance can only be negative for *dQ* < 0.

While negative capacitance is a rather well known effect in light emitting and laser diodes [26, 27, 37], the peak accompanying the drop in capacitance, seen in Figure 6(a), was not reported previously. Negative *dQ* results from a combination of various recombination processes. These include mainly a high rate of radiative recombination and non-radiative processes originating from charge trapping in interfacial states at both boundaries of the intrinsic layer [26–28]. A second reason for NC accompanied by a sharp peak is penetration of charges stored in the QW reservoir layer to the QDs in accordance with the damped resonant tunnelling model [38]. The peak value of the negative capacitance and the shape of the peak strongly depend on the damping constant and therefore can vary over a wide range.

**Figure 7:**
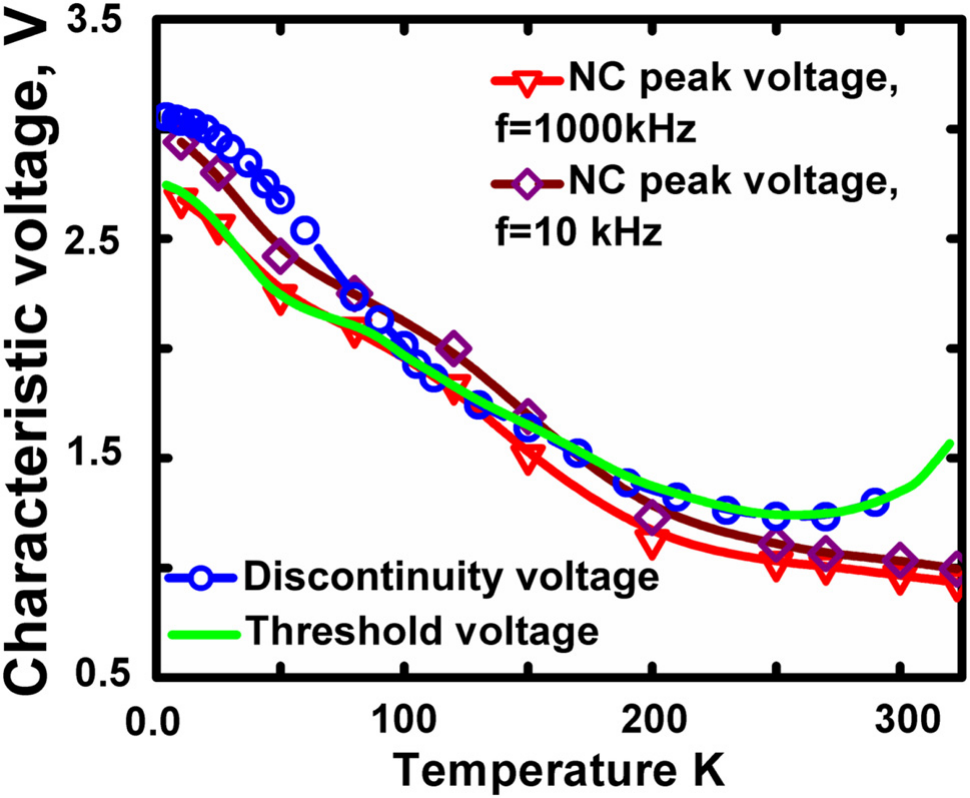
Temperature dependence of the characteristic voltages.

The NC sharp narrow peak is related to the discontinuity near 3 V in the *α*–*V* curve and the peaks in the *γ*–*V* characteristics. This correlates the various electrical and electro-optical properties and proves that the electro-optical characteristics have a clear imprint on the electrical properties. The correlations are determined via the temperature dependence of characteristic voltages corresponding to the measured data as described in Figure 7.

In the temperature range of 4 K–60 K, the main threshold voltage equals the peak NC voltage at 1000 kHz. This means that the onset of stimulated emission, and the corresponding tunnelled carrier injection from the QW, as well as the carrier clamping, induce the change in charge density that causes the NC. In this temperature range, the discontinuity in *α*–*V*, which represents the second threshold occurs at a higher voltage as is the 10 kHz NC peak. Around 100 K, the two threshold voltages merge at the value that corresponds to the NC peak at 1000 kHz. At temperatures up to 150 K, the NC peak voltage at 10 kHz is higher than that at 1000 kHz. This stems from the fact that some traps induce slow non-radiative processes which cannot be sensed at the higher frequency. In the range of 150–200 K, the characteristic voltages at both frequencies are almost the same, but are lower than that threshold voltage. Non-radiative recombination increases above 200 K due to enhancement of phonon induced processes [39], which leads to a decrease in efficiency and an increase in the threshold voltage.

## 3 Conclusions

In summary, we have presented a comprehensive study of the relationship between optical and electrical characteristics of TI QD lasers. At low temperatures we observed, in the *α*–*V* characteristics, discretization of the various dot layers as well as a periodic response, both of which are a direct signature of the resonant tunnelling process that feeds carriers from the QW reservoir to the QD ground state via hybridization with a QD excited state. The QD gain inhomogeneity causes, below 60 K, emission at two separate wavelength regimes. These have two different threshold voltages which have a clear imprint on the *α*–*V*, *γ*–*V* as well as the *C*–*V* characteristics. The latter show hysteresis loops with a temperature dependent width. At the voltage regime where the laser reaches threshold, the *C*–*V* curves exhibit an inductive nature which represents NC. At a measurement frequency of 1000 kHz, the NC peak voltage, at temperatures up to 150 K, occurs at a voltage corresponding to the onset of stimulated emission. At 10 kHz, the NC is associated with non-radiative recombination. Above 150 K, non-radiative recombination plays a significant role which naturally leads to reduction of the laser efficiency.
